# New Diterpenoids from the Aerial Parts of *Salvia reuterana*

**Published:** 2019

**Authors:** Mahdi Moridi Farimani, Mansour Miran, Samad Nejad Ebrahimi

**Affiliations:** a *Department of Phytochemistry, Medicinal Plants and Drugs Research Institute, Shahid Beheshti University, G.C., Evin, Tehran, Iran. *; b *Department of Pharmacognosy, Faculty of Pharmacy, Tehran University of Medical Sciences, Tehran, Iran.*

**Keywords:** *Salvia reuterana*, Labdane diterpene, Structure elucidation, NMR, Cytotoxicity

## Abstract

The genus *Salvia *is a valuable origin of structurally diverse terpenoids. In a project directed at structurally interesting bioactive metabolites from Iranian *Salvia *species, we studied *Salvia reuterana.* Two new labdane diterpene, 6β, 14α-dihydroxy-15-acetoxysclareol (1), and 14α, 15- dihydroxy sclareol (2), were isolated from the aerial part of the plant. Their structures were established mainly by 1D and 2D NMR spectroscopic techniques, including ^1^H-^1^H COSY, HSQC and HMBC methods and HR-ESI-TOFMS spectral data. Compound 1 and 2 were tested for their inhibitory activity toward HeLa and MCF-7 cell lines. Our results showed that *S. reuterana* is a rich source of labdane diterpenoids. These compounds are rather rare in *Salvia *species, although they are frequently found in other genera of the *Lamiaceae*. *S. reuterana* is a new source of these diterpenoids.

## Introduction

*Salvia*, consisting of about 1000 species, is the largest genus in the family *Lamiaceae* and widely distributed in various regions of the world, Namely, the Mediterranean area, south Africa, central and south America, and southeast Asia (1). Plant of the genus *Salvia *have attracted much attention owing a variety of medicinal properties and biological activities, such as antibacterial, antioxidant, to antitumor, anti-diabetic and anti-Inflammatory activities (1-3). The Iranian ﬂora comprises 61*Salvia* species, 17of which are endemic (4). Some Iranian *Salvia *species have been investigated from a phytochemical viewpoint, and antibacterial, antiprotozoal, and anticancer compounds have been reported (5-12.). S*alvia reuterana *Boiss. is an endemic species which grows in the highlands of central Iran (13). Its common name in Persian is “Mariam Goli Esfahani” (14), and the aerial parts of the plant are traditionally used as sedative and anxiolytic herbal medicine. In addition, the antibacterial, antioxidant, free radical scavenging and anti-anxiety properties of this herb have been proved in recent studies (15–19). In a previous work, we have reported the presence of six labdane diterpenoids from this plant (20). Further examination of the *n*-hexane extract of *S. reuterana* led to the isolation of two new other labdane diterpenoids (1, 2). 

## Experimental

*General*

HR-ESI-MS was carried out on a Bruker 147 microTOF–ESIMS system. NMR spectra were recorded on Bruker AVANCE III-500 spectrometer operating at 500.13 MHz for ^1^H NMR and 125.77 MHz for ^13^C NMR with TMS as an internal standard. Silica gel (70-230 and 230-400mesh) used for column chromatography and silica gel F_254_ (20×20 cm) for TLC, were both supplied by the Merck company.

*Plant material*

The aerial parts of *Salvia reuterana *were collected from the northern hilly areas of Tehran in Juley 2011. A voucher specimen (MPH-1321) has been deposited in the herbarium of Medicinal Plants and Drugs Research Institute (MPH) of Shahid Behshti University, Tehran, Iran.

*Extraction and Isolation*

The air-dried aerial parts of *S. reuterana* (3.0 kg) was crushed and extracted with n-hexane (3 × 15 L( by maceration at room temperature. Extract was concentrated in *vacuo*, to afford 130g of a dark gummy residue. The residue was separated on a silica gel column (230-400 mesh, 850g) with a gradient of n-hexane-EtOAc (100/0 to 0/100) as eluent, followed by increasing concentration of MeOH (up to 25%) in EtOAc. On the basis of TLC analysis, fractions with similar composition were pooled to yield 27 combined fractions.

Fractions 22, 23 and 24 were combined (2g) and subjected to silica gel column chromatoghraphy (70-230 mesh), eluted with CHCl_3_-Me_2_CO-MeOH (77:13:10) to give six subfraction (C_1_-C_6_). Subfraction C_2 _was rechromatographed over silica gel (70-230), eluted with CHCl_3_-MeOH (1:1), to afford three subfraction (C_21_-C_23_). Subfraction C_21_ was recrystallized from CHCl_3_ to afford compound **1** (3.5mg). Fraction 25 (3g) was applied to a silica gel column chromatography (70-230, mesh), eluted with Me_2_CO-hexane (2:1) to separate eight subfraction (f_1_-f_8_). Subfraction f_2_ was recrystallized from hexane to afford compound **2** (8mg).

*6β, 14α-dihydroxy-15-acetoxysclareol* (1): White powder; [α] = -9.6 (c 0.2, CHCl_3_); for ^1^H and ^13^C NMR data, see Table 1; HR-ESI-TOFMS *m/z*423.2738 [M + Na] ^+^, calcd for 400.2802).

*14α, 15-dihydroxysclareol* (2): White powder; [α]= -7.1(c 0.2, CHCl_3_); for ^1^H and ^13^C NMR data, see Table 1; HR-ESI-TOFMS *m/z*365.2712 [M + Na] ^+^, calcd for 342.5107).

**Table 1:** 1H and 13C NMR data of compounds 1 and 2 in CDCl /CD OD (500 MHz for δ ; 125 MHz for δ )

	**1**		**2**	
**position**	**δ** **C**	**δ** **H ** **(J in Hz)**	**δ** **C**	**δ** **H** **(J in Hz)**
1α 1β	41.6	0.85^b^ 1.52^b^	40.0	0.88^b^ 1.53^b^
2α 2β	18.3	1.60^b^ 1.33^b^	18.4	1.53^b^ 1.35^b^
3α 3β	44.1	1.05^b^ 1.22^b^	41.8	1.07^b^ 1.28^b^
4	33.4		32.9	
5α	56.7	0.83^b^	56.7	0.83^b^
6α 6β	67.6	4.39 brd (2.6)	20.6	1.57^b^ 1.18^b^
7α 7β	51.3	1.55^b^ 1.91 dd (13.5, 2.0)	43.8	1.34^b^ 1.75 dt (12.5,3.0)
8	73.4		74.5	
9α	62.1	1.03^b^	62.3	1.00 t (3.5)
10	38.9		39.2	
11α 11β	17.9	1.49 1.28	18.0	1.46^b^ 1.26^b^
12α 12β	40.9	1.60^b^ 1.41^b^	40.8	1.60^b^ 1.38^b^
13	73.1		73.8	
14β	75.1	3.55 dd (8.5, 2.5)	77.8	3.39 dd (7.5, 3.5)
15a 15b	66.2	3.96 dd (11.5, 8.5) 4.22 dd (11.5, 2.5)	62.5	3.50 dd (11.5, 7.5) 3.65 dd (11.5, 3.5)
16	22.6	1.10 s	22.0	1.08 s
17	24.8	1.32 s	23.6	1.09 s
18	32.8	0.89 s	33.4	0.79 s
19	24.2	1.10 s	21.5	0.71 s
20	16.1	1.08 s	14.9	0.73 s
**C**H3CO CH3**C**O	19.8 171.3	1.99 s		

a δ values were extracted from COSY, HSQC and HMBC experiments.

b Overlapping signals.

**Table 2:** Cytotoxic activities of compounds 1 and 2 and against two human tumor cell lines

**Compound**	**Hela (IC** **50 ** **μM)**	**MCF-7 (IC** **50** **μM)**
1	184.61 ± 1.67	108.34 ± 2.07
2	218.47 ± 2.77	164.07 ± 3.09
Paclitaxel^b^	0.004	0.033

a Data are expressed as mean ± standard deviation (n = 3).

b Positive control.

**Figure 1:**
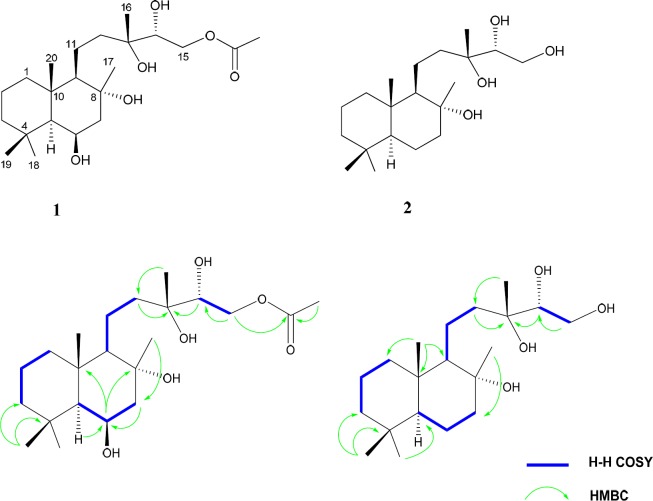
Key H–H COSY and HMBC connectivities of compounds **1 **(left) and **2 **(right)

*Cytotoxicity assay*

The human epithelioid cervix carcinoma (Hela) and human breast adenocarcinoma (MCF-7) cell lines were purchased from National Cell Bank of Iran (NCBI), Pasteur Institute of Iran (Tehran, Iran), and maintained in DMEM medium supplemented with 10% fetal bovine serum and 100 U/mL penicillin and 100 μg/mL streptomycin. These cells were kept at 37 °C in a humidified atmosphere containing 5% CO_2_. Compounds 1-3 were dissolved in DMSO to make a stock of 1mg/mL and further diluted to final concentrations of 10-100 μg/mL with the serum free culture medium.

*Cell viability assay*

Cell viability was determined using the MTT assay. Briefly, 2.5×104 cells were seeded in 96-well plates at 37 °C with 5% CO_2_ for overnight incubation and treated with appropriate concentrations of compounds 1-3 for 24 h. The cells were then incubated with a serum-free medium containing MTT at a final concentration of 0.5 mg/mL for 4 h. The dark formazan crystals formed were dissolved in DMSO and the absorbance was measured at 570 nm.

## Results and Discussion

Compound **1** was obtained as a white amorphous powder. Its HI-ESI-TOFMS exhibited a molecular ion peak at m/z 423.2738 [M + Na^+^], agreed with the molecular formula C_22_H_40_O_6_. The molecular formula accounted for three degrees of unsaturation. The ^13^C NMR spectrum (Table 1), showed 22 carbon resonances which were analyzed from the DEPT-HSQC spectra to consist of six methyls, seven methylens, four methines and four quaternary carbons. Two carbon signals at δ_C _73.1 and 73.4 indicated the presence of oxygen bearing sp^3^quaternary carbons. Another oxygenated carbons at δ_C _75.1 and 67.6 accounted for the carbons carrying two hydroxyl groups with the carbinolic methine protons coming at δ_H_ 3.55 (1H, dd, *J *= 2.5, 8.5 Hz) and δ_H_ 4.39 (1H, brd, *J*= 2.6 Hz), respectively. The signal at δ_C _66.2 accounted for amethylenic carbon attached to an acetoxy group, with the corresponding methylene protons coming at 3.96, 4.22 each as a doublet of doublet. According to the degree of unsaturation the molecule was bicyclic and appeared to be a labdane diterpenoid (21). The ^1^H and ^13^C NMR spectrum displayed features similar to those of 14*α*-hydroxy-15-acetoxysclareol**, **isolated previously from this plant by us (20). However, the ^13^C NMR spectrum of **1** showed the presence of an additional methine at *δ*_C _67.6 instead of the methylene group (C-6). Hence, the methylene was replaced by an oxygenated methine. The signals of C-7 (*δ*_C_51.3) and C-5 (*δ*_C_56.7) were paramagnetically shifted (Δ*δ* +7.3 and +0.7 ppm, respectively) in comparison to those of 14*α*-hydroxy-15-acetoxysclareol. Also the resonances of neighboring H-7*α *(*δ*_H_ 1.55), H-7𝛽 (*δ*_H_ 1.91), and H-5*α* (*δ*_H_ 0.83) were shifted (Δ*δ*= +0.2, +0.15, and -0.03ppm, respectively). HMBC correlations between H-5*α*, H-7*α* with C-6, and between H-6, C-8, and C-10 (Figure 1) confirmed the location of the hydroxyl group. Diagnostic COSY correlations were observed between H-6 and H-7*α*and H-7*β*, and between H-6 and H-5. The relative configuration of the hydroxyl group at C-6 was determined as *β *based on the magnitude of the vicinal coupling constants of the H-6 resonance (*δ*_H_ 4.39*, *brd, *J *= 2.6 Hz). NOESY contacts of H-6 with H-5 and H-7ax were observed. The ^1^H chemical shifts of CH_3_-17, CH_3_-19 and CH_3_-20 (*δ*_H_ 1.32, 1.10 and 1.08, respectively) appeared downfield (*δ*_H_ 0.21, 0.38, and 0.35) relative to those of 14*α*-hydroxy-15-acetoxysclareol. These differences were in agreement with an axial orientation of the hydroxyl group at C-6. Thus, the structure of **1 **was established as 6*β*,14*α*-dihydroxy-15-acetoxysclareol.

Compound **2 **was obtained as a white powder. The HR-ESI-TOFMS showed a peak at m/z 365.2712 [M+Na] ^+^, in agreement with the elemental formula C_20_H_33_O_4_, which accounted for three degrees of unsaturation. The ^1^H and ^13^C NMR data (Table 1) strongly resembled those of **1**, indicating that the two compounds were structurally related. Inspection of the ^1^H and ^13^C NMR spectra showed the lack of the signals belonges to the acetoxy group in compound **2.** Also, the methine signals at67.6 ppm (δ_H_ 4.39) was replaced by a methylene group (δ_C _20.6 and δ_H_ 1.57, 1.18) in **2**. Thus, compound **2 **was established as 14α, 15- dihydroxy sclareol.

Compounds **1** and **2** were evaluated for their *in vitro* cytotoxic activity against Hela (human epitheloid cervix carcinoma) and MCF-7 (human breast adeno-carcinoma) cell lines. The results of the cytotoxicity studies were indicated in Table 2.

## Conclusion

Our results showed that *S. reuterana* is a rich source of labdane diterpenoids. The most abundant diterpenoids in the genus are abietanes and rearranged abietanes. Labdane diterpenoids are rather rare in *Salvia* species although they are frequently found in other genera of the *Lamiaceae*. *S. reuterana* is a new source of these diterpenoids. 

## References

[1] Pan ZH, Wang YY, Li MM, Xu G, Peng LY, He J, Zaho Y, Li Y, Zaho QS (2010). Terpenoids from Salvia trijuga. J. Nat. Prod.

[2] Nickavar B, Rezaee J, Nickavar A (2016). Effect-directed analysis for the antioxidant compound in Salvia verticillata. Iran. J. Pharm. Res.

[3] Firuzi OR, Miri R, Asadollahi M, Eslami S, Jassbi AR (2013). Cytotoxic, Antioxidant and antimicrobial activities and phenolic contents of eleven Salvia species from Iran. Iran. J. Pharm. Res.

[4] Jamzad Z, Assadi M, Maassoumi A, Mozaffarian V (2012). Lamiaceae. Flora of Iran.

[5] Abedini A, Roumy V, Mahieux S, Gohari S, Moridi Farimani M, Riviere C, Samaillie J, Sahpaz S, Bailleul F, Neut C, Hennebelle T (2014). Antimicrobial activity of selected Iranian medicinal plants against a broad spectrum of pathogenic and drug multi-resistant micro-organisms. Lett. Appl. Microbiol.

[6] Ebrahimi SN, Moridi Farimani M, Mirzania F, Soltanipoor MA, De Mieri M, Hamburger M (2014). Manoyloxide sesterterpenoids from Salvia mirzayanii. J. Nat. Prod.

[7] Jassbi AR, Zare S, Firuzi OR, Xiao J (2016). Bioactive phytochemicals from shoots and roots of Salvia species. Phytochem. Rev.

[8] Moridi Farimani M, Matloubi-Moghaddam F, Esmaeili MA, Amin GA (2012). Lupane triterpenoid and other constituents of Salvia eremophila. Nat. Prod. Res.

[9] Moridi Farimani M, Mazarei Z (2014). Sesterterpenoids and other constituents from Salvia lachnocalyx Hedge. Fitoterapia.

[10] Moridi Farimani M, Abbas-Mohammadi M, Esmaeili MA, Salehi P, Ebrahimi SN, Sonboli A, Hamburger M (2015). Seco-ursane-type triterpenoids from Salvia urmiensis with apoptosis-inducing activity. Planta Med.

[11] Moridi Farimani M, Taleghani A, Aliabadi A, Aliahmadi A, Esmaeili MA, Sarvestani NN, Khavasi HR, Smiesko M, Hamburger M, Ebrahimi SN (2016). Labdane diterpenoids from Salvia leriifolia: Absolute configuration, antimicrobial and cytotoxic activities. Planta Med.

[12] Moridi Farimani M, Abbas-Mohammadi M (2016). Two new polyhydroxylated triterpenoids from Salvia urmiensis and their cytotoxic activity. Nat. Prod. Res.

[13] Rechinger KH (1987). Flora Iranica. Austria. Graz.

[14] Mozaffarian V (1996). A dictionary of Iranian plant names.

[15] Salimpour F, Mazooji A, Darzikolaei SA (2011). Chemotaxonomy of six Salvia species using essential oil composition markers. J. Med. Plant Res.

[16] Rabbani M, Sajjadi SE, Jafarian A, Vaseghi G (2005). Anxiolytic effects of Salvia reuterana Boiss on the elevated plus-maze model of anxiety in mice. J. Ethnopharmacol.

[17] Ghomi, JS, Masoomi R, Kashi FJ, Batooli H (2012). In-vitro bioactivity of essential oils and methanol extracts of Salvia reuterana from Iran. Nat. Prod. Commun.

[18] Esmaeili, MA, Kanani MR, Sonboli A (2010). Salvia reuterana extract prevents formation of advanced glycation end products: An in-vitro study. Iran. J. Pharm. Sci.

[19] Esmaeili A, Rustaiyan A, Nadimi M, Larujani K, Nadjafi F, Tabrizi L, Chalabian F, Amiri H (2008). Chemical composition and antibacterial activity of essential oils from leaves, stems and flowers of Salvia reuterana Boiss. grown in Iran. Nat. Prod. Res.

[20] Moridi Farimani M, Miran M (2014). Labdane diterpenoids from Salvia reuterana. Phytochemistry.

[21] Su YA, Kazuo K, Dean G, Jia J, Liu J, Zheng J, Tamotso N (2002). Three new labdane diterpenoid glycosides from Conyza blinii. Heterocycles.

